# Prevalence of comorbidities and associated factors in acromegaly patients in the Turkish population

**DOI:** 10.3906/sag-2007-243

**Published:** 2021-06-28

**Authors:** Mustafa CAN, Muhammet KOCABAŞ, İlker ÇORDAN, Hatice ÇALIŞKAN BURGUCU, Melia KARAKÖSE, Mustafa KULAKSIZOĞLU, Feridun KARAKURT

**Affiliations:** 1 Division of Endocrinology and Metabolism, Department of Internal Medicine, Faculty of Medicine, Necmettin Erbakan University, Konya Turkey

**Keywords:** Acromegaly, comorbidity, growth hormone, insulin-like growth factor 1

## Abstract

**Background/aim:**

The presence of comorbidities in patients with acromegaly causes an increase in morbidity and/or mortality and a decrease in quality of life. In this study, we aimed to investigate the demographic, clinical and laboratory features, prevalence of acromegaly-related comorbidities, and factors associated with these comorbidities in patients with acromegaly.

**Materials and methods:**

In the study, 96 patients who were followed up with the diagnosis of acromegaly were included. Clinical, laboratory and imaging features, and accompanying comorbidities of the patients were recorded from the patient files.

**Results:**

Of the patients included in the study, 63 (65.6%) were female and 33 (34.4%) were male. The mean age of diagnosis was 42.61± 12.08, and the mean follow-up period was 9.97 ± 7.26 years. Median insulin-like growth factor 1 level was 238.16 ng/mL (30.5–820), median growth hormone level was 2.05 ug/L (0.1–29.4). A total of 60 (62.5%) of the patients were in the well-controlled group, and 36 (37.5%) had active disease at the time of inclusion. Diabetes mellitus (DM) was detected in 30 (31.3%) patients, prediabetes in 19 (28.8%) patients, hypertriglyceridemia in 38 (42.2%) patients, hypertension (HT) in 41 (42.7%) patients, cardiovascular disease in 5 (5.2%) patients, malignancy in 9 (9.4%) patients, obstructive sleep apnea syndrome in 8 (8.3%) patients, carpal tunnel syndrome in 11 (11.5%) patients, arthropathy in 5 (5.2%) patients, hearing loss in 7 (7.3%) patients, thyroid nodule in 56 (67.5%) patients, thyroid cancer in 4 (4.2%) patients, colonic polyp in 19 (38.8%) patients.

**Conclusion:**

In this study, we revealed that the most common comorbidities in acromegaly patients in the Turkish population are thyroid nodules, low high-density lipoprotein (HDL cholesterol (HDL-C) level, hypertriglyceridemia, HT, colonic polyps, DM, and prediabetes, and female sex and age at diagnosis are the most important factors associated with comorbidities.

## 1.Introduction

Acromegaly is a rare, chronic disease characterized by increased growth hormone (GH) and insulin-like growth factor 1 (IGF-1) levels, mostly caused by GH secreting pituitary adenoma.

Although acromegaly is a rare disease, its associated comorbidities and the required lifetime management mean that acromegaly can be a large burden [1]. Long-term presence of increased GH and IGF-1 levels in acromegaly is associated with complications, such as diabetes mellitus (DM), cardiovascular disease (CVD), obstructive sleep apnea syndrome (OSAS), increased mortality especially due to CVD and cancer, impaired quality of life, and low life expectancy [2]. Since the time from onset of symptoms to diagnosis is approximately 4.5 to 5 years in acromegaly, complications are common both during and after diagnosis.

In this study, we aimed to investigate the demographic, clinical, laboratory and imaging features, disease activity status, therapies they received, the prevalence of comorbidities, and the factors associated with comorbidities in patients followed up with the diagnosis of acromegaly.

## 2.Materials and methods

96 patients over 18 years old who were followed up with the diagnosis of acromegaly between January 2006 and January 2020 in the Necmettin Erbakan University School of Medicine Department of Endocrinology were included in the study. Necmettin Erbakan University, School of Medicine Ethics Committee approved the study with the approval number of 2020/2511 and the date of 22.05.2020.

The demographic, clinical, laboratory data of the patients were recorded from the patient files. Patients with a family history of cancer, especially thyroid and colon cancer, and a history of radiotherapy to the head and neck region for reasons other than acromegaly were excluded from the study.

A normal IGF-1 level in terms of age and sex and lack of suppression of GH to 0.4 ng/mL following documented hyperglycemia during an oral glucose load or random GH level <1 ng/mL were determined as criteria for being included in the “well-controlled” group. Those who did not meet these criteria were included in the “active disease” group. 

The imaging features of the patients evaluated by magnetic resonance imaging (MRI) were recorded from their files. Pituitary adenomas less than 10 mm in diameter were considered as microadenomas, adenomas with a diameter of 10 mm and larger were considered as macroadenoma. Patients who met any of the following criteria were considered to have DM: fasting plasma glucose (FPG) ≥ 126 mg/dL or 75 gram oral glucose tolerance test (OGTT) 2nd h plasma glucose ≥ 200 mg/dL or random plasma glucose ≥ 200 mg/dL in a person with diabetes symptoms or HbA1C≥6.5%. Patients with FPG between 100–125 mg/dL (ımpaired fasting glucose-IFG) or OGTT 2nd h PG between 140–199 mg/dL (ımpaired glucose tolerance-IGT) or HbA1C 5.7-6.4% were considered to have prediabetes. Patients with triglyceride ≥ 150 mg/dL were considered to have hypertriglyceridemia, and patients with high density lipoprotein cholesterol (HDL-C) < 40 mg/dL for men and HDL-C < 50 mg/dL for women were considered to have low HDL-C. Lesions larger than 3 mm were considered as nodules in thyroid ultrasonography (US). Patients whose nerve conduction study/electromyography findings were consistent with CTS were considered to have carpal tunnel syndrome (CTS). Patients with cardiovascular disease proven by methods, such as electrocardiography, echocardiography and coronary angiography, were considered to have CVD. Patients whose polysomnograhy results were compatible with OSAS were considered to have OSAS. Those determined to have conductive, sensorineural, or mixed type hearing loss as a result of audiometry measurements were considered to have hearing loss. Those who had arthropathy confirmed by imaging methods (X-ray, MRI) but did not have other arthropathy causes (rheumatoid arthritis, psoriatic arthritis etc.) were considered to have arthropathy.

### 2.1.Statistical analysis

Statistical analysis were performed using the SPSS 22.0 (IBM Corp., Armonk, NY, USA) program. Continuous variables were given as mean ± standard deviation when the distribution was normal and median (minimum-maximum) when it was nonnormal. To compare the independent group differences, the significance test of the difference between the two means (independent samples t test) was used if there were parametric test assumptions; if there are no parametric test assumptions, the Mann–Whitney U test was used. Pearson’s correlation coefficient was used for correlation analysis between numerical variables with normal distribution, and Spearman test was used for correlation analysis between numerical variables that did not show normal distribution. The chi-squared test was used to evaluate the differences between categorical variables. For differences, P value <0.05 was considered statistically significant.

## 3.Results

Of the patients included in the study, 63 (65.6%) were female and 33 (34.4%) were male. The mean age of the patients was 42.61 ± 12.08 years at the time of the diagnosis of the acromegaly, and the median follow-up was 9.97 ± 7.26 years. The median IGF-1 level was 238.16 ng/mL (30.5–820), and the median GH level was 2.05 ug/L (0.1–29.4) (Table 1). A total of 33 of the patients had microadenoma and 63 had macroadenoma. Two patients had hyperprolactinemia accompanying acromegaly.

**Table 1:** Demographic characteristics, complications and comorbidity prevalence of patients according to groups.

	All acromegaly population	Active disease group	Well-controlled group	P
Age of diagnosis (years)	42.61± 12.08	43.51 ± 12.66	42.10 ± 11.81	0.230
Sex (female/male)	63/33	23/13	40/20	0.977
Follow-up time (years)	9.97 ± 7.26	9,83± 8,02	10.05 ± 6.85	0.390
IGF-1 (ng/mL)	238.16 (30.5–820)	402.07 (202.9–820)	144.94(30.5–332.4)	< 0.001
GH (ug/L)	2.05 (0.1–29.4)	4.02 (0.6–29.4)	0.918 (0.1–7.9)	< 0.001
Adenom size (micro/macro)	33/63	8/28	25/35	0,023
DM n (%)	30/96 (31.3)	16/36 (44.4)	14 (23.3)	0.007
Prediabetes n (%)	19/66 (28.8)	8/20 (40.0)	11 (23.9)	0.851
Hypertriglyceridemia n (%)	38/90 (42.2)	16/35 (45.7)	22/55 (40.0)	0.480
Low HDL-C n (%)	44/90 (48.9)	15/35 (42.9)	29/55 (52.7)	0.480
HT n (%)	41/96 (42.7)	16/36 (44.4)	25/60 (41.7)	0.437
CVD n (%)	5/96 (5.2)	2/36 (5.6)	3/60 (5)	0.993
Malignancy n (%)	9/96 (9.4)	3/36 (8.3)	6/60 (10)	0.871
OSAS n (%)	8 /96 (8.3)	4/36 (11.1)	4/60 (6.7)	0.882
CTS n (%)	11/96 (11.5)	3/36 (8.3)	8/60 (13.3)	0.355
Arthropathy n (%)	5/96 (5.2)	0 (0.0)	5/60 (8.3)	0.149
Hearing loss n (%)	7/96 (7.3)	3/36 (8.3)	4/60 (6.7)	0.447
Thyroid nodule n (%)	56/83 (67.5)	23/32 (71.9)	33/51 (64.7)	0.476
Thyroid cancer n (%)	4/83 (4.2)	1/32 (2.8)	3/51 (5.9)	0.577
Colonic polyp n (%)	19/49 (38.8)	8/20(40)	11/29 (37.9)	0.960

A total of 93 patients had undergone surgical treatment, and medical treatment was started as the primary treatment modality in 3 patients who refused surgical treatment. Radiotherapy (RT) was applied to 29 patients and 14 (48.3%) of the patients who received radiotherapy were in remission. Forty-six patients were on medical treatment at the time of inclusion, of which 27 were on somatostatin receptor ligand (SRL) and 19 were on cabergoline treatment. 9 (33.3%) of 27 patients who were on SRL were in remission, and 6 (31.6%) of 19 patients who were on cabergoline were in remission. A total of 60 (62.5%) patients were in the well controlled group, and 36 (37.5%) patients had active disease.

Of the 36 patients in the active disease group, 23 were female and 13 were male. The mean age at diagnosis of 36 patients in the active disease group was 43.51 ± 12.66, and the mean follow-up period was 9.83 ± 8,02 years. The median IGF-1 level in the active disease group was 402.07 ng/mL (202.9–820), and the median GH level was 4.02 ug/L (0.6–29.4) (Table 1). A total of 8 of the patients had microadenoma and 28 had macroadenoma. In the active disease group, 16 (44.4%) patients were on SRL, 5 (13.9%) patients were on cabergoline, and 1 (2.8%) was on SRL plus cabergoline.

Of the 60 patients in the well controlled group, 40 were female and 20 were male. The mean age at diagnosis of 60 patients in the well controlled group was 42.10 ± 11.81, and the mean follow-up period was 10.05 ± 6.85 years. The median IGF-1 level in the well controlled group was 144.94 ng/mL (30.5–332.4), and the median GH level was 0.918 ug/L (0.1–7.9) (Table 1). A total of 25 of the patients had microadenoma and 36 had macroadenoma. 

In terms of metabolic complications in all acromegaly patients, 30 (31.3%) patients had DM, 19 (28.8%) patients had prediabetes, 38 (42.2%) patients had hypertriglyceridemia, and 44 (48.9%) patients had low HDL-C levels. As cardiovascular complications, 41 (42.7%) patients had HT and 5 (5.2%) patients had CVD (atrial fibrillation in 2 patients, coronary artery disease in 2 patients, cardiomyopathy in 1 patient). Nine (9.4%) patients had malignancy and papillary thyroid cancer observed in 4 (4.2%) patients was the most common malignancy. And there were one (1.04%) patient with each diagnosis of myelodysplastic syndrome, basal cell carcinoma, lymphoma, meningioma, and renal cell carcinoma. In terms of other comorbidities, 8 (8.3%) patients had OSAS, 11 (11.5%) patients had carpal tunnel syndrome (CTS), 5 (5.2%) had arthropathy, and 7 (7.3%) had hearing loss. Thyroid nodules were detected in 56 (67.5%) of 83 patients who underwent thyroid US. Thyroid cancer was detected in 4 (4.2%) patients. Colonic polyp was detected in 19 (38.8%) of 49 patients who underwent colonoscopy. 4 (4.2%) of the patients had hypopituitarism (2 panhypopituitarism, 1 central hypothyroidism plus central hypogonadism, 1 central hypogonadism) in the preoparative period due to the mass effect of adenoma (Table 1). Twenty-five patients were on antidiabetic, 12 patients were on antilipidemic, 38 patients were on antihypertensive medication.

In active disease group, IGF-1 level (P < 0.001), GH level (P < 0.001), presence of macroadenoma (P = 0.023) and presence of DM diagnosis (P = 0.02) were higher than well controlled group (Table 1). Other demographic parameters and complications were similar between groups.

In the correlation analysis, we found correlations between DM and age at diagnosis (r = –0.309, P = 0.002), female sex (r = 0.204, P = 0.046) and having active disease (r = 0.236, P = 0.020); between low HDL-C and female sex (v -0.385, P = 0.001); between hypertension and age at diagnosis (r = -0.551, P = 0.001), female sex (r: 0.270, P = 0.008) and adenoma size (r = –0.218, P = 0.033); between OSAS and age at diagnosis (r = –0.266, P = 0.009); between malignancy and female sex (r = 0.203, P = 0.047); between thyroid nodule and age at diagnosis (r = –0.307, P = 0.005); between colonic polyp and age at diagnosis (r = –0.367, P = 0.011) (Table 2). There was no significant relationship between the duration of the disease and the frequency of comorbidities (Table 2).

**Table 2:** Correlation analysis of parameters associated with the prevalence of comorbidities.

	DM	Low HDL-C	HT	OSAS	Malignancy	Thyroid nodule	Colonic polyp
Age of diagnosis	r = –0.309P = 0.002	r = 0.099P = 0.355	r = –0.551P = 0.001	r = –0.266P = 0.009	r = 0.014P = 0.889	r = –0.307P = 0.005	r = –0.367P = 0.011
Follow-up time	r = 0.015P = 0.877	r = –0.035P = 0.740	r = 0.098P = 0.341	r = 0.110P = 0.285	r = –0.142P = 0.167	r = 0.010P = 0.927	r = –0.086P = 0.565
Female sex	r = 0.204P = 0.046	r = –0.385P = 0.001	r = 0.270P = 0.008	r = –0.050P = 0.628	r = 0.203P = 0.047	r = 0.050P = 0.654	r = –0.232P = 0.117
Active disease	r = 0.236P = 0.020	r = 0.008P = 0.938	r = –0.046P = 0.656	r = 0.037P = 0.718	r =– 0.046P = 0.657	r = 0.055P = 0.624	r = 0.028P = 0.151
Adenom size	r = –0.127P = 0.217	r = 0.123P = 0.247	r = –0.218P = 0.033	r = 0.034P = 0.740	r = 0.119P = 0.250	r = –0.136P = 0.219	r = –0.214P = 0.149

## 4.Discussion

In this study, we found that the most common comorbidities in acromegaly patients in the Turkish population are thyroid nodule, low HDL-C level, hypertriglyceridemia, HT, colonic polyp, DM, and prediabetes. Our study revealed that the most important factors associated with comorbidities are female sex and age at diagnosis; only DM is more common in the active disease group than the well-controlled group, and the frequencies of other comorbidities are similar between the groups.

Glucose metabolism disorders, such as DM and prediabetes are common in patients with acromegaly. Presence of DM in acromegaly patients is an important predictive factor for increased mortality [3]. In a retrospective study, Kreze et al. reported the DM prevalence as 19% and the impaired glucose tolerance prevalence as 5%. The authors also found that the risk factors associated with DM and impaired glucose tolerance are family history of DM and HT, female sex, concomitant prolactin hypersecretion, and untreated active disease [4]. In another retrospective study, Biering et al. found DM prevalence as 40.5%, impaired glucose tolerance prevalence as 28.2% and reported that DM is more common in female patients [5]. In our study, the prevalence of DM was 31.3%, the prevalence of prediabetes was 28.8%, and we showed that the risk factors for DM are female sex, age at diagnosis, having active disease. In our study, we also showed that DM prevalence is more frequent in the active disease group than in the well-controlled group.

Increased circulating levels of lipoprotein-A, apolipoprotein-A1, and apolipoprotein-E as a result of insulin resistance in acromegaly leads to disorders of lipoprotein metabolism. The most common lipid metabolism disorders in acromegaly are hypertriglyceridemia and low HDL-C level. Ciresi et al. found the prevalence of hypertriglyceridemia as 33.2% and the prevalence of low HDL-C as 39.1%, and also found that the prevalence of low HDL-C is higher in women (especially postmenopausal) than men [6]. Møller et al. identified compensatory hyperinsulinemia as a risk factor associated with hypertriglyceridemia [7]. In our study, hypertriglyceridemia prevalence was 42.2%, low HDL-C prevalence was 48.9%, and sex was found as the factor associated with low HDL-C. 

HT is one of the most common cardiovascular comorbidities in patients with acromegaly. In a metaanalysis of 18 series including 2562 patients, the prevalence of hypertension was reported to be around 35% [8]. Jaffrain-Rea et al. showed that the risk factors associated with HT are the degree of glucose tolerance abnormalities and the age of the patient [9]. Other cardiovascular complications that can be seen in patients with acromegaly are cardiomyopathy, valvulopathy, arrhythmias, and atherosclerotic cardiovascular diseases caused by vascular endothelial dysfunction. Symptomatic CVD is seen in approximately 20% of patients with acromegaly [10]. In our study, we found the prevalence of HT as 42.7% and the prevalence of CVD as 5.2%. Additionally, the risk factors associated with HT were sex, age at diagnosis, and adenoma size.

Respiratory complications associated with acromegaly usually include sleep apnea, sleep respiratory disorders, and respiratory failure [11]. Causes of respiratory complications associated with acromegaly include decreased lung flexibility, changes in respiratory mucosa and cartilage, anatomical abnormalities that affect craniofacial bones and soft tissues. Acromegaly is generally associated with obstructive sleep apnea syndrome (OSAS) arising from anatomical abnormalities. In a prospective study, Davi et al. found the prevalence of sleep apnea in the entire acromegaly population, in the group with active disease, and in the group in which the disease was in remission, respectively, as 47%, 56%, and 39%. The authors also reported that the factors associated with sleep apnea were age, male sex, IGF1, body mass index (BMI), and disease duration [12]. In our study, we found that the prevalence of OSAS is 8.3%, the factor associated with OSAS is age at diagnosis.

There are several clinical studies and animal studies showing that high IGF-1 and GH levels are associated with cancer development and progression [13]. In most studies, patients with acromegaly have been reported to have a moderately increased risk for developing thyroid, colon, breast, hematological, and prostate cancer. In retrospective studies in patients with acromegaly, the prevalence of malignancy was found to be 6.3 to 14% [14,15]. In a prospective study, Baris et al. reported that gastrointestinal tract (small intestine, colon), brain, thyroid, kidney, and bone cancers are the most common cancer types in acromegaly [14]. In a retrospective study, Kurimoto et al. reported that colon, thyroid, breast, and gastric cancer are the most common in patients with acromegaly [15]. In our study, we found the malignancy prevalence as 8.3% and was similar to previous studies. However, unlike previous studies, we found thyroid cancer as the most common cancer type in our study. We found female sex as the only risk factor associated with malignancy.

 A common complication associated with acromegaly is CTS. The main cause of median nerve neuropathy in patients with acromegaly is edema of the median nerve in the carpal tunnel [16]. Oktayoglu et al. reported the prevalence of CTS as 50% in patients with acromegaly [17]. In our study, the prevalence of CTS was found to be 11.5%, and we did not determine a risk factor associated with CTS.

Arthropathy is common in patients with acromegaly. In acromegaly patients, arthropathy is thought to be due to two mechanisms, initially GH and IGF-1 excess, and later mechanical changes [18]. In a prospective study of Kropf et al., the prevalence of arthropathy was reported to be 56% in patients with acromegaly [19]. They found increased BMI, advanced age, and female sex as factors associated with arthropathy. In our study, we found the prevalence of arthropathy as 5.2%, and we did not determine a risk factor associated with arthropathy.

Hearing loss is common in patients with acromegaly. Although the cause of hearing loss in acromegaly is not fully known, it is thought to be caused by soft tissue enlargement and bony hypertrophy. Aydin et al. performed a prospective study of acromegaly patients; hearing loss was found to be 43% [20]. In our study, we found the prevalence of hearing loss as 7.3% and we did not determine a risk factor associated with hearing loss.

Thyroid nodules are common in patients with acromegaly. IGF-1 stimulates protein and deoxyribonucleic acid synthesis in tyrosides and leads to cell proliferation and differentiation [21]. Prolonged exposure to high serum IGF-1 levels in acromegaly is thought to lead to the development of thyroid nodules [22]. In the meta-analysis of Wolinski et al., thyroid nodule and thyroid cancer prevalences were reported as 59.2% and 4.3%, respectively [21]. In retrospective studies in patients with acromegaly, the prevalence of thyroid cancer has been reported to be 1 to 3% [22]. In our study, we found thyroid nodule and thyroid cancer prevalences as 67.5% and 4.2%, respectively. In our study, we found age at diagnosis as factors related to thyroid nodules. We did not find a factor associated with thyroid cancer.

Both benign and malignant colon lesions are frequently encountered in patients with acromegaly. Prolonged exposure to high GH and IGF-1 levels is thought to play a role in the pathogenesis of the development of colon lesions [23]. In addition, it is thought that the continuous high levels of GH and IGF1 may facilitate the growth of pre-existing colon tumors [24]. Kurimoto et al. retrospective study reported that colon polyp prevalence was 40.2%, and colon cancer prevalence was 10.3% in patients with acromegaly [15]. Another retrospective study reported the prevalence of hyperplastic polyp, adenomatous polyp and colon cancer in patients with acromegaly as 19.1%, 23.4%, and 4.3%, respectively [25]. In our study, the prevalence of colonic polyp (38.8%) was similar to previous studies. In our study, we found age at diagnosis as a factor related to colonic polyp. In our study, there was no patient with colon cancer.

When we compare our results with previous studies, we noticed that hypertriglyceridemia, low HDL-C, and thyroid nodule prevalences were higher than previous studies, DM, prediabetes, HT, malignancy, and colonic polyp prevalences were similar to previous studies, while CVD, OSAS, CTS, arthropathy, and hearing loss prevalences were lower than previous studies.

Our study had some limitations. First, our study population was limited. Secondly, since our study was a retrospective study, not all complications were evaluated in all patients. Presumably due to these limitations, the prevalences of comorbidities, such as DM, prediabetes, hypertriglyceridemia, low HDL-C, HT, malignancy, thyroid nodule, colonic polyp were similar to or lower than previous studies, whereas prevalences of comorbidities such as CVD, OSAS, CTS, arthropathy, and hearing loss were very low. 

In conclusion, regular follow-up of all complications, particularly malignancies, metabolic, and cardiovascular complications, is required in all patients to improve morbidity and mortality in acromegaly patients.

**Acknowledgments/disclaimers/conflict of interest**

The authors declare that there is no conflict of interest between the authors of the article. The authors declared that they did not receive any financial support in this study.

None of the authors have any material interest in any of the products, devices or medicines mentioned in this article. The research was not supported by an external organization. All authors agreed to allow full access to primary data and to allow the journal to review the data if desired.
